# Tobacco consumption behavior change during the COVID-19 pandemic is associated with perceived COVID threat

**DOI:** 10.1186/s12889-024-20259-5

**Published:** 2024-10-15

**Authors:** Hollyann F. Loui, Joshua Li, Nicholas J. Jackson, Ruby Romero, Lauren E. Wisk, Russell G. Buhr

**Affiliations:** 1 Division of Pulmonary & Critical Care Medicine, David Geffen School of Medicine at the University of California, Los Angeles, 1100 Glendon Avenue, Suite 850, Los Angeles, CA 90024 USA; 2 Department of Medicine Statistics Core, David Geffen School of Medicine at the University of California, Los Angeles, Los Angeles, CA USA; 3 Division of General Internal Medicine & Health Services Research, David Geffen School of Medicine at the University of California, Los Angeles, Los Angeles, CA USA; 4 Department of Health Policy and Management, Jonathan and Karin Fielding School of Public Health at the University of California, Los Angeles, CA USA; 5 Center for the Study of Healthcare Innovation, Implementation, and Policy, Health Systems Research, Greater Los Angeles Veterans Affairs Healthcare System, Los Angeles, CA USA

**Keywords:** Tobacco, Smoking, Vaping, COVID-19, Mental health

## Abstract

**Rationale:**

Tobacco use is a risk factor for COVID-19 adverse outcomes. Despite health implications, data conflict regarding COVID-19 and tobacco consumption. We present results from a survey of health behaviors during the pandemic to identify how COVID-19 influenced tobacco behaviors.

**Methods:**

A nationally administered, internet-based survey was deployed between May–September 2020. Of respondents, we analyzed participants who reported current smoking and/or vaping. Our primary outcome of interest was change in tobacco or vape use using measures from the Behavioral Risk Factor Surveillance System, as well as whether participants reported that these changes were related to COVID-19. Our principal exposures were previously psychometrically evaluated measures of anxiety, depression, and novel perceived COVID-19 threat scale with additional adjustment for age. We employed multinomial logistic regression to determine associations between these factors and tobacco consumption.

**Results:**

We identified 500 respondents who reported ever smoking in their lifetime, 150 of which reported currently smoking at the time of the survey. Of 220 participants who reported any use of vapes, 110 reported currently vaping. Increased perceived threat of COVID-19 was associated with both increased (aRR_increase_ 1.75, 95% CI [1.07–2.86], *P* = 0.03) and decreased (aRR_decrease_ 1.72 [1.04–2.85], *P* = 0.03) tobacco consumption relative to no change. There were no significant relationships found between perceived threat of COVID-19 and vaping behavior.

**Conclusions:**

As perceived COVID-19 threat increased, people were more likely to increase or decrease their smoking as opposed to continue at the same amount of use, even after controlling for anxiety and depression, both of which are known to affect smoking in either direction. Further study into motivators of changing tobacco consumption behaviors, and how barriers to care from safer-at-home policies and changes in care delivery moderate change in tobacco use will aid planning tobacco reduction interventions during the ongoing and future respiratory viral pandemics.

**Trial registration:**

This manuscript is derived from baseline survey data obtained in the “Understanding Community Considerations, Opinions, Values, Impacts, and Decisions in COVID-19” study. ClinicalTrials.gov registration NCT04373135, registered 04/30/2020.

**Supplementary Information:**

The online version contains supplementary material available at 10.1186/s12889-024-20259-5.

## What is already known on this topic

- Prior to this study, there was little data about how tobacco consumption had changed in the setting of the COVID-19 pandemic, and the data that did exist conflicted.

## What this study adds

- This study examined tobacco and vaping behavior changes during the COVID-19 pandemic.
- We found that perceived threat of COVID-19 had a significant impact on increasing tobacco use and almost significant impact on decreasing tobacco use even after controlling for anxiety and depression, making tobacco reduction interventions and strategies even more integral as the pandemic continues.

## How this study might affect research, practice, or policy

- Recognizing that worsening mental health and perceived threat of COVID-19 both have negative impacts on tobacco use is critical for effective tobacco cessation strategies for the ongoing and future pandemics.

## Introduction

Tobacco use and tobacco-related diseases such as cardiovascular disease and chronic obstructive pulmonary disease are risk factors for severe COVID-19 infection and adverse outcomes [1–3]. Despite these known health implications, data conflict regarding the impact of the COVID-19 pandemic on tobacco consumption globally, both for traditional and electronic cigarettes. Recent research has shown mixed results: while some studies point to a trend toward increased tobacco use during the COVID-19 pandemic, others suggest decreased usage [4–8].

COVID-19 was a new, strong motivator to decrease tobacco use, and higher perceived risk of COVID-19 is associated with increased interest in tobacco cessation [5, 6]. Those who deliberately decreased tobacco consumption during the early COVID-19 pandemic cited concerns about contracting COVID-19 and becoming severely ill due to smoking’s link to lung damage, fear of spreading COVID-19 to others, or a desire to be healthier as their motivation to reduce or quit smoking [4, 5, 9]. Even those reporting low knowledge about smoking as a risk factor for COVID-19 disease severity were incentivized to quit or reduce consumption due to the social restrictions COVID-19 placed on tobacco use: safer-at-home policies limited smoking with friends, at parties, and when publicly socializing [7, 8].

Desire to reduce or quit tobacco use do not always match outcomes. A US study examining post-hospitalization tobacco cessation interventions that collected data between May and July 2020 found that almost half of participants (41%) who had been smoking in January 2020 (pre-COVID-19-pandemic) reported increased interest in reducing or quitting smoking since the start of the pandemic. Although all study participants expressed some intention to quit smoking, a third of respondents ended up increasing their consumption when surveyed again months later, which correlated with higher perceived stress [5]. This finding matches reports of increased cigarette sales during the pandemic [10, 11]. While COVID-19 presented a strong case for improving overall health, it has also been a significant source of psychological distress, which may account for increasing tobacco use, at least in part due to the positive association between smoking and depression and anxiety [12]. In an Italian survey, higher perceived stress levels were associated with increased smoking, in addition to other factors reflective of worsening mental health including decreased sleep, decreased quality of life, increased depression, and increased anxiety [13, 14]. Likewise, other risk factors for tobacco use include unemployment, lower income, and alcohol consumption [13].

Identifying and understanding how COVID-19 has influenced tobacco use could be useful in understanding how acute exogenous stress events such as disasters or pandemics influence tobacco use behavior, fortifying our understanding of how to develop effective cessation strategies. Reducing tobacco use in the general population would elevate public health and decrease the global disease burden of many illnesses including COVID-19. In this manuscript, we present the results from a nationally administered survey that included questions about health behavior changes during the COVID-19 pandemic, with the hypothesis that COVID-19 influenced participants to change their tobacco smoking and vaping behaviors.

## Methods

### Recruitment of participants

The survey was disseminated through snowball sampling using social media platforms, as well as via direct recruitment through community partners and advocacy organizations, and medical professional societies, referenced in the acknowledgements. Detailed methodology on the design, deployment, and recruitment of participants was previously published [15]. Though our recruitment efforts were enriched within California related to additional survey modules outside the scope of this report, eligibility was not restricted by location and all adults (age ≥ 18) were eligible. Notably, within California, approximately 10.9% of residents use tobacco [16], as opposed to 11.5% nationally [17]. Recruitment for this wave of the survey opened on May 8, 2020 and closed on September 30, 2020. This study was approved by the University of California Los Angeles Institutional Review Board (20–000683) and registered with ClinicalTrials.gov (NCT04373135). Informed consent was obtained from all participants at enrollment before completing the first wave survey and all methods were carried out in accordance with relevant guidelines and regulations.

### Survey design

We drafted and deployed an internet-based survey to engage community opinions on how COVID-19 had affected participants’ ability to work, socialize, maintain their health, and seek medical care, as well as sociodemographic information to inform our analyses. Survey questions were deployed on REDCap version 10.6.14 (Vanderbilt University, Nashville, TN) [18, 19] initially. They were subsequently professionally translated (International Contact, Berkeley, CA) into Spanish, Korean, Mandarin, Vietnamese, and Tagalog and migrated to Qualtrics XM (Qualtrics, LLC, Provo, UT) for multilingual support, which was not available in REDCap. This survey was part of a larger study contained 4 relevant sections to this report, and 2 sections outside the scope of this manuscript. Section 1 contained 20 sociodemographic items. Section 2 contained 22 health status and health behavior items including smoking and vaping and mental health status items. Section 3 contained 9 items on access to health care services. Section 4 contained 22 items on the impact of COVID-19 on respondents, including perceived threat from COVID-19.

### Survey items of interest

Of interest for this report were sociodemographics, smoking and vaping related behaviors, perceived threat from COVID-19, and participant-reported anxiety or depression. Smoking and vaping items were taken directly from the Centers for Disease Control and Prevention’s Behavioral Risk Factor Surveillance System, which contains validated and widely used items on tobacco use [20, 21]. For smoking, participants were first asked if they had “smoked at least 100 cigarettes, or if not cigarettes, 20 cigars, 40 bowls of pipe tobacco, or 1 h of hookah cumulatively in their life.” For vaping, participants were asked if they had “ever used e-cigarettes or vapes in their entire life. E-cigarettes are electronic devices that usually contain a nicotine-based liquid that is vaporized and inhaled. You may also know them as vape-pens, hookahpens, electronic hookahs (e-hookahs), electronic cigars (e-cigars), electronic pipes (e-pipes), or e-vaporizers. Some look like cigarettes, and others look like pens or small pipes. These are battery-powered devices that produce vapor instead of smoke.” For those who answered yes to either of these questions, for each there were subsequent items. Next, they were asked “Do you now smoke [and/or vape] every day, some days, or not at all?”. Respondents were considered “currently” smoking [or vaping] if they answered “some days” or “every day”. Because our items mirrored BRFSS, we did not further stratify by product type. Respondents were then asked “In the past 30 days, have you changed how much you smoke [and/or vape] for any reason?” with response categories of “Yes—I started smoking [or vaping] more”, “No—I have not changed my smoking [or vaping]” or “Yes—I started smoking [or vaping] less”. Finally, respondents who indicated change to smoking [or vaping] indicated whether this change was related to COVID-19.

The anxiety scale used was the Generalized Anxiety Disorder-2 (GAD-2) questionnaire with a score of greater than 3 used to define a positive screen for anxiety [22, 23]. For depression, the Patient Health Questionnaire-2 (PHQ-2) was used with a score greater than 3 used to define a positive screen for depression [24].

A novel “Perceived Coronavirus Threat Questionnaire” scale developed by Conway and colleagues was used to determine associations with respondents’ concern about COVID. This six-item scale was graded on a 7-point Likert scale of the following items: (1) “Thinking about the coronavirus (COVID-19) makes me feel threatened.” (2) “I am afraid of the coronavirus (COVID-19).” (3) “I am not worried about the coronavirus (COVID-19)”, which is reverse-scaled. (4) “I am worried that I or people I love will get sick from the coronavirus (COVID-19).” (5) “I am stressed around other people because I worry I’ll catch the coronavirus (COVID-19).” (6) “I have tried hard to avoid other people because I don’t want to get sick.” The psychometric properties of this scale are detailed by the authors in a working paper [25]. For our analyses, the items in the novel scale were collapsed using standard factor analysis, such that a weighted scale score was tabulated from the individual questions and centered upon the mean, where each point on the scaled battery equals 1 standard deviation difference in weighted score compared to the sample mean [15].

### Statistical analysis

We focused this report on those who reported any lifetime tobacco or vaping use as defined above, further restricting the analytic sample to the subset of participants who reported that they currently smoke or use an e-cigarette/vape at the time they completed the survey. Bivariate analysis examined self-reported change over the past 30 days in each of smoked or vaporized tobacco use (increased, decreased, no change) with depression and anxiety scores (PHQ-2, GAD-2), the perceived COVID-19 threat scale score, and baseline characteristics, using Analysis of Variance (ANOVA) for continuous variables and Chi-squared tests for discrete variables.

Multivariable multinomial logistic regression models using complete case analysis were fit to examine how perceived COVID-19 threat was associated with change in smoked or vaporized tobacco use after adjusting for other confounding variables (i.e. PHQ-2 or GAD-2 scores). A new composite binary variable measuring a high PHQ-2 or GAD-2 score (either score > 3) was created due to collinearity when PHQ-2 and GAD-2 scores were treated as separate variables. Demographics including age, gender, education, and work were excluded from our statistical models to conserve statistical power in the setting of the small sample size and lack of significant association in bivariate analyses, but were included for description of the cohort.

### Missing data

We compared an unadjusted model using only COVID-19 threat to an adjusted model including age and mental health for our primary analyses. Because ~ 28% of respondents reporting current smoking had at least one missing datapoint on a covariate of interest for our regression models due to attrition in progressively later sections in the survey, we first utilized sensitivity analyses that incorporated an inverse probability weight (IPW) to account for factors associated with having missing data. This IPW was created using a logistic regression model for complete (1) vs missing (0) data based on these predictors: the presence of PHQ-2/GAD-2 scores, the participant’s change in mental health, and gender.

In a second set of sensitivity analyses for missing data, we used multiple imputation using chained equations Multivariate Imputation by Chained Equations (MICE) with m = 10 imputations. Values were imputed for COVID-19 Threat Scale Score, PHQ-2 Total Score, and GAD-2 Total Score based on tobacco (or vape) use change, age, gender, and the imputed variables (COVID-19 Threat, PHQ-2, GAD-2). Binary versions of the PHQ and GAD were then created based on these imputed values for use in models (where applicable). As outlined in prior biostatistical work, comparing the two techniques, IPW was less likely to have biased estimates, but MICE was more likely to be precise [26]. All of the statistical analyses and modeling was done in R 4.2.1 with use of the nnet and ggplot2 packages [27, 28].

## Results

### Cohort demographics

**Fig. 1:**
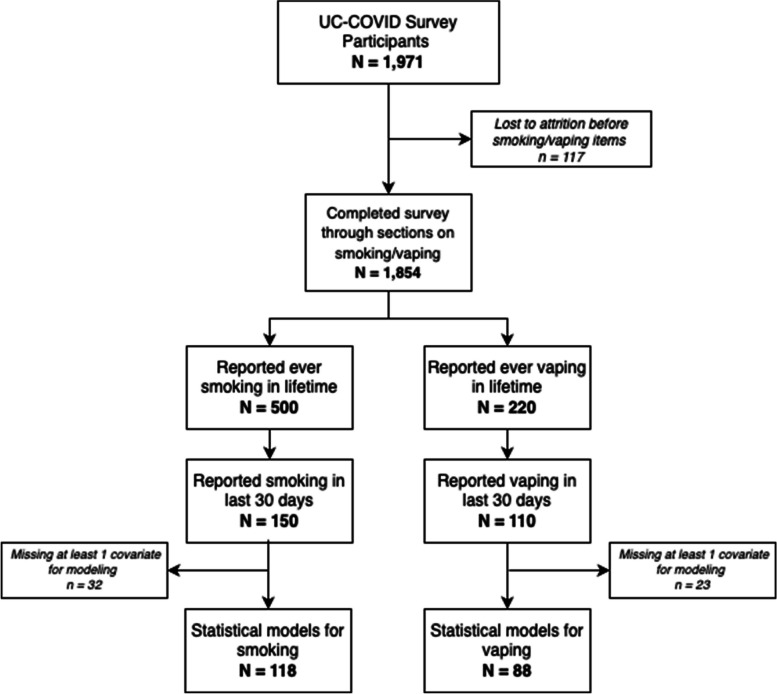
CONSORT diagram illustrating flow of survey respondents into our analyses

We analyzed participants who reported any lifetime use of tobacco products (combusted tobacco or e-cigarettes/vapes), of whom 500 (88%) reported any lifetime cigarette use and 220 (39%) reported any lifetime e-cigarette/vape use (Fig. 1). Additionally, 48 respondents reported both smoking and vaping at least some days in the 30 days prior to participation in the survey. Of these, 150 individuals reported current cigarette use, of whom 59% identified as female with a mean ± SD age of 51 ± 15 years. Among the 220 participants who reported any lifetime use of vapes or e-cigarettes, 110 participants reported currently vaping, among whom 45% identified as female, with an average age of 41 ± 13 years.

### Change in tobacco smoking behavior

A majority (56%) of respondents changed their smoking behavior, and of those who changed 60% reported smoking more than prior while 40% smoked less. Respondents who decreased smoking were, on average, 4 years older than those that did not change smoking and 9 years older than those who smoked more (*P* = 0.04, Table 1). We did not observe statistically significant differences in smoking behavior across self-reported gender identity, race, ethnicity, education, or employment in our respondents (Table 1). Those who screened positive for depression by PHQ-2 score > 3 were almost twice (64% vs. 36%) as likely to increase smoking. Those who screened positive for anxiety by GAD-2 score > 3 were three times (76% vs. 24%) as likely to smoke more and slightly less (41% vs. 59%) inclined to smoke less than non-anxious counterparts in bivariate analyses (*P* = 0.03). Because there did not appear to be a significant relationship between smoking changes and demographics including gender, race/ethnicity, education, and work status in our bivariate analyses, these variables were not adjusted for in our final models.

**Table 1:** Characteristics of respondents who reported smoking or vaping and change thereof during the pandemic

	**Smoking (*N* = 150)**				**Vaping** ** (*N* = 110)**			
	**No Change** **(*n* = 66)**	**Smoking More** **(*n* = 50)**	**Smoking Less** **(*n* = 34)**	***P*-value**	**No Change** **(*n* = 54)**	**Vaping More** **(*n* = 26)**	**Vaping Less** **(*n* = 30)**	***P*-value**
**Frequency of use last 30 days, n (%)**				⊗				⊗
***Some days***	25 (38%)	19 (38%)	8 (24%)		19 (35%)	14 (54%)	4 (13%)	
***Every day***	36 (54%)	31 (62%)	12 (35%)		35 (65%)	12 (46%)	18 (60%)	
*Never (quit in last 30d)*	0 (0%)	0 (0%)	14 (41%)		0 (0%)	0 (0%)	7 (23%)	
*Did not report*	5 (8%)	0 (0%)	0 (0%)		0 (0%)	0 (0%)	1 (3%)	
**Age in years,** mean (SD)	46 (16)	41 (13)	50 (17)	0.04	43 (15)	40 (13)	46 (16)	0.39
**Sex,** n (%)								
*Female*	39 (59%)	27 (54%)	22 (65%)	0.62	27 (50%)	8 (31%)	15 (50%)	0.22
*Male*	27 (41%)	23 (46%)	12 (35%)		27 (50%)	18 (69%)	15 (50%)	
**Race/Ethnicity,** n (%)								
*Not White*	11 (18%)	8 (16%)	9 (29%)	0.32	14 (26%)	2 (8%)	7 (24%)	0.18
*White*	51 (82%)	42 (84%)	22 (71%)		40 (74%)	23 (92%)	22 (76%)	
**Education Level,** n (%)								
*Associate’s*	16 (24%)	12 (24%)	12 (35%)	0.60	12 (22%)	3 (12%)	3 (10%)	0.26
*Bachelor’s*	21 (32%)	20 (40%)	11 (32%)		22 (41%)	8 (31%)	15 (50%)	
*Graduate Level*	29 (44%)	18 (36%)	11 (32%)		20 (37%)	15 (58%)	12 (40%)	
**Employment,** n (%)								
*Working*	33 (50%)	34 (68%)	21 (62%)	0.14	39 (72%)	21 (81%)	21 (70%)	0.62
*Not Working*	33 (50%)	16 (32%)	13 (38%)		15 (28%)	5 (19%)	9 (30%)	
**COVID Threat Scale Score,** mean (SD)	-0.47 (1.08)	0.04 (0.88)	0.06 (0.84)	0.02	-0.2 (1.04)	-0.11 (0.74)	0.21 (0.59)	0.17
**PHQ-2 Score,** mean (SD)	2.1 (1.8)	3.5 (1.7)	2.0 (2.0)	< 0.01	2.2 (2.1)	2.8 (2.1)	2.0 (1.4)	0.38
**Depression + Screen,** n (%)								
*No*	37 (59%)	13 (36%)	22 (67%)	0.03	27 (60%)	11 (61%)	20 (69%)	0.72
*Yes*	26 (41%)	23 (64%)	11 (33%)		18 (40%)	7 (39%)	9 (31%)	
**GAD-2 Score,** mean (SD)	2.3 (1.9)	3.8 (1.7)	2.5 (2.1)	< 0.01	2.8 (2.2)	3.8 (1.7)	2.6 (1.6)	0.04
**Anxiety + Screen,** n (%)								
*No*	37 (57%)	11 (24%)	20 (59%)	< 0.01	25 (48%)	7 (28%)	16 (53%)	0.14
*Yes*	28 (43%)	34 (76%)	14 (41%)		27 (52%)	18 (72%)	14 (47%)	

N.B.: *PHQ-2* = Patient Health Questionnaire, 2 items; *GAD-2* = Generalized Anxiety Disorder Questionnaire, 2-items; COVID Threat Scale = factor weighted perceived threat to COVID-19 (novel scale) where each point change is 1 SD change in participant response compared to mean of sample. *P*-values are ANOVA for continuous items and Chi squared tests for categorical items

^⊗^ *P*-value not calculated as groupings inherent to inclusion criteria and are different by definition

In our unadjusted model, each standard deviation increase above the survey population mean perceived threat of COVID-19 measured by the novel COVID-19 threat scale score was significantly associated with both increased (RR_increase_ 1.71, 95% CI [1.07, 2.74]) and decreased (RR_decrease_ 1.77 [1.07, 2.92]) tobacco consumption relative to no change suggesting a U-shaped relationship between perceived COVID threat and smoking behaviors (Table 2). After controlling for the presence of either depression or anxiety using an aggregate variable that combined screening positive for anxiety or depression based on GAD-2 and PHQ-2, respectively, and age, we still found significant relationships between both increased (aRR_increase_ 1.75, 95% CI [1.07, 2.86]) and decreased (aRR_decrease_ 1.72, 95% CI [1.04, 2.85]) smoking behavior and perceived threat of COVID-19 (Table 2).

**Table 2:** Change in smoked tobacco use based on perceived threat of COVID

**Tobacco Change**	**Unadjusted** **(*N* = **118**)**		**Adjusted** (*N* = 116)	
	**Relative Risk** **(95% CI)**	***P*-value**	**Relative Risk (95% CI)**	***P*-value**
**COVID Threat Scale Score**				
*More v No Change*	1.71 (1.07, 2.74)	0.03	1.75 (1.07, 2.86)	0.03
*Less v No Change*	1.77 (1.07, 2.92)	0.03	1.72 (1.04, 2.85)	0.03
**Age**				
*More v No Change*			1.00 (0.97, 1.03)	0.96
*Less v No Change*			1.00 (0.97, 1.03)	0.93
**Anxiety or depression positive screen (either)**				
*More v No Change*			3.21 (1.13, 9.12)	0.03
*Less v No Change*			0.54 (0.2, 1.47)	0.22

**Fig. 2:**
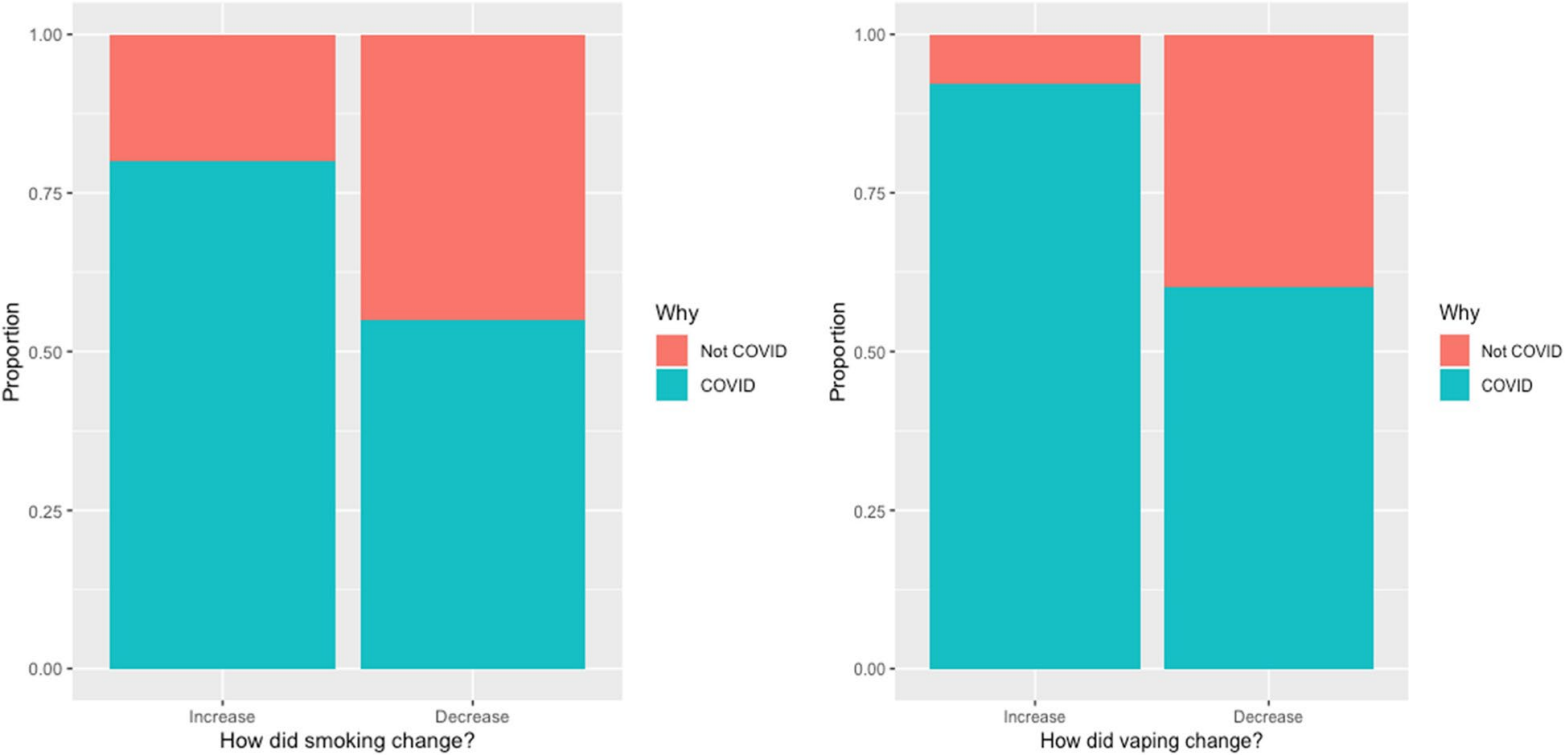
Self-reported change in smoking behavior and whether the change was influenced by COVID-19. Change in smoking behavior was reported by respondents as more likely to be due to COVID-19 than not (for both increased and decreased use)

When examining how smoking behavior changed, and when asked directly whether the change was influenced by COVID-19, participants self-reported that their change in smoking behavior was more likely to be due to COVID-19 than not for both increased and decreased use. For smoking, 80% of those who smoked more and 55% of those who smoked less responded that the change was due to COVID-19. For vaping, COVID-19 was cited as the reason for change among 92% those vaping more and 60% of those vaping less (Fig. 2).

### Change in vaping behavior

Slightly more participants (51%) changed their vape or e-cigarette use than remained the same, and among those that changed, more respondents reported decreasing their use (53%) than increasing (47%). In our bivariate analyses, only anxiety symptoms were associated with change in vaping behavior, where those who reported increase in vaping having a 1.2 point higher score on the GAD-2 than those who decreased their vaping and 1 point higher than those who did not change the amount they vape. Interestingly, depression did not have a significant relationship with vaping change. We did not observe significant relationships between self-reported vaping changes and demographics including age, gender identity, race/ethnicity, education, and work status. In our regression models, we similarly observed no statistically significant relationship between perceived COVID-19 threat and vaping behaviors in either unadjusted or adjusted analyses (Table 3).

**Table 3:** Change in vaping behavior based on perceived threat of COVID and other variables

**Vaping Change**	**Unadjusted** **(*N* = **88**)**		**Adjusted** (*N* = 87)	
	**Relative Risk** **(95% CI)**	***P*-value**	**Relative Risk** **(95% CI)**	***P*-value**
**COVID Threat Scale Score**				
*More v No Change*	1.11 (0.61, 2.02)	0.73	1.03 (0.55, 1.95)	0.93
*Less v No Change*	1.87 (0.96, 3.67)	0.07	1.89 (0.97, 3.68)	0.06
**Age**				
*More v No Change*			0.99 (0.95, 1.03)	0.60
*Less v No Change*			1.00 (0.96, 1.03)	0.89
**Anxiety or depression positive screen (either)**				
*More v No Change*			1.34 (0.42, 4.28)	0.62
*Less v No Change*			0.72 (0.26, 2.02)	0.53

### Sensitivity analyses

In inverse probability weighted (IPW) models, we observed a minimally augmented effect of COVID-19 perceived threat on smoking increase, with decrease in smoking falling just below statistical significance. There was no observed change in sign in any covariate for our smoking models. No significant changes in effect were noted in the vaping analyses in the IPW models (Supplemental Tables 1 and 2). Findings were parallel for imputed models (Supplemental Tables 3–4).

## Discussion

Our results demonstrated that perceived threat from COVID-19 is associated with change in smoked tobacco use, observed as both increased and decreased smoking. Based on this, we hypothesize that concern around COVID-19 likely influenced people to change their tobacco behaviors, even after accounting for depression and anxiety, which is an area for further exploration in future work. Although we were not able to find statistically significant relationships between worsening mental health and vaping behaviors, a similar pattern was observed*.* These trends may have been present but were possibly limited by the statistical power of the study as there were fewer respondents who reported vape or e-cigarette use when compared to those who reported smoking as their method of tobacco use.

Fear of COVID-19 provides a strong incentive for decreased tobacco consumption: smoking is linked to lung damage which is a risk factor for COVID-19 disease severity, contracting the illness comes with concern for potentially spreading it to others, the social pressure from others to quit or reduce smoking, and a general desire to be healthier in the face of a pandemic, among other reasons [1, 2, 5]. Moreover, social restrictions from safer-at-home policies and fear of contracting the illness from others limited venues for smoking, and the financial burdens of the pandemic may have forced many individuals to quit the expensive habit out of necessity [4–9, 29]. Others who were more social smokers may have unknowingly cut down on their tobacco use due to social restrictions from stay-at-home policies, and therefore never intended to quit. Our items did not directly address this possibility, which warrants additional research.

Interestingly, our study found that perceived threat of COVID-19 showed slightly larger effect size for increasing consumption of tobacco products than decreasing, albeit with overlapping confidence intervals that suggest against statistically significant differences in relative risk. Many studies have demonstrated a clear positive association between smoking and mental illness, likely corresponding here to the psychological stress of COVID-19 and the resultant anxiety, depression, and overall worsening mental health associating with increased tobacco and vaping use [12]. Historically, stress-provoking events with state-level, national, or global impact like the terrorist attack of September 11, 2001 have been linked to increased tobacco use, and the COVID-19 pandemic has had significant negative impact worldwide [13]. In addition, the COVID-19 pandemic saw increased rates of unemployment and alcohol consumption, and drops in income: all risk factors for increased tobacco usage in other studies [13, 14, 30]. As such, the complex social, biological, and psychological dynamics associated with the COVID-19 pandemic likely had myriad effects on respondents, including influencing their tobacco behaviors to change, whether in a positive or negative direction.

Stress is linked to increased difficulty quitting tobacco, and the concomitant stress of the COVID-19 pandemic and challenge of fighting an addiction (smoking and/or vaping) likely posed a huge barrier to successful cessation for many users of vape and tobacco products [4]. Varenicline, a medication used for tobacco cessation, was subject to a nationwide voluntary recall by Pfizer in July 2021. Though beyond the time horizon for this analysis, given this issue, we expect that ongoing changes related to tobacco behaviors may have been further compounded by this recall and potentially posed yet another hurdle to successful tobacco reduction and cessation during the ongoing pandemic [31].

Prior to the pandemic, tobacco industry research found that younger people who smoke of higher education and income were more likely to reduce or quit smoking [9, 32]. Generally, younger people who smoke are more successful at reducing tobacco consumption and quitting than their older counterparts. The pandemic contributed to even higher quit rates amongst younger people who smoke who were no longer able smoke with their friends at school or at bars or clubs, reported that mask wearing made smoking inconvenient, or lived with parents unaware of their smoking habit and therefore were forced to quit during pandemic-related restrictions [7]. In contrast, in our study younger respondents were more likely to increase smoking and vaping, corresponding with an alarming uptick in tobacco use among younger adults over the past 20 years [33].

At the same time we saw that older respondents were more likely to decrease use. While we did not collect the necessary data to further explain this, it is possible that older participants viewed their personal risk from COVID-19 as being higher than younger counterparts, providing additional incentive to quit. This finding serves to generate hypotheses for future study on the complex interplay of demographic factors and external stressors that we were underpowered to explore in this analysis. Our results did not show a significant relationship between tobacco behaviors and work, education, or self-reported gender. These trends may have been present but were possibly limited by the statistical power of the study, potentially contributing to type 2 error.

### Limitations

This study recruited through community-engagement and social media methods. While perhaps less generalizable than national probability samples, this method of recruitment is still known to generate valuable insights and may be particularly useful during a pandemic when recruitment to studies is more challenging in general [34]. The possibility of non-response bias remains, as we have detailed in other manuscripts from this overall study, though the completion rate through the section that included smoking was 94% of those who began the survey [15]. The small sample size precluded us from robustly evaluating subgroups who may be at increased or decreased risk for changing smoking behavior. The survey did not monitor changes in tobacco or vape use over multiple time points, precluding measurement of the impact of the pandemic on tobacco use as the pandemic progressed. Additionally, while the items on smoking and vaping change were taken from BRFSS, a widely used and highly studied method of assessing health behaviors, this decision meant we did not assess things like change in quantity of combusted tobacco smoked per day, change to concentration of vaping solution, move from smoking to vaping, or other potential changes of interest. Regardless, our findings serve to support the existing knowledge base on behavior change in smoking and vaping and generate hypotheses on the relationships between exogenous stressful events and tobacco use and its impact on tobacco control efforts.

## Conclusion

Prior to our study, there was limited data on how the COVID-19 pandemic had influenced tobacco use, and the existing data appeared to conflict. On one hand, latent effects of the pandemic, such as economic stress, may have led people to increase their smoking during COVID-19. While conversely, smoking was considered a risk factor for worse COVID-19 outcomes, possibly motivating people to reduce consumption of tobacco. However, neither outcome was universally observed across the sample. Our study adds some clarity to the complex relationship between COVID-19 and tobacco use behaviors, finding that increasing perceived COVID-19 threat had a U-shaped relationship with smoking: people were more likely to increase or decrease their smoking than stay the same. This increase in tobacco consumption leaves us with pressing research questions: was information about smoking risk not received, was it not resonating with them for some reason, and how do interruptions in usual routines, both in health care and social settings undermine cessation efforts, all of interest to public health in efforts to reduce tobacco use.

## Supplementary Information

Supplementary Material 1: Supplemental Table 1. Change in smoked tobacco use based on perceived threat of COVID with inverse probability weighting for complete responses. Supplemental Table 2. Change in vaping behavior based on perceived threat of COVID with inverse probability weighting for complete responses. Supplemental Table 3. Change in smoked tobacco use based on perceived threat of COVID using multiple imputation. Supplemental Table 4. Change in vaping behavior based on perceived threat of COVID using multiple imputation.

## Data Availability

The datasets used and/or analyzed during the current study are available from the corresponding author on reasonable request.
