# Supplementary material for: Tobacco consumption behavior change during the COVID-19 pandemic is associated with perceived COVID threat
Source: BMC Public Health. 2024 Oct 15;24:2827. doi: 10.1186/s12889-024-20259-5 (PMC11476555; doi:10.1186/s12889-024-20259-5)
Supplement: Supplementary file 1 — Supplementary Material 1: Supplemental Table 1. Change in smoked tobacco use based on perceived threat of COVID with inverse probability weighting for complete responses. Supplemental Table 2. Change in vaping behavior based on perceived threat of COVID with inverse probability weighting for complete responses. Supplemental Table 3. Change in smoked tobacco use based on perceived threat of COVID using multiple imputation. Supplemental Table 4. Change in vaping behavior based on perceived threat of COVID using multiple imputation. [file 12889_2024_20259_MOESM1_ESM.docx]

**Tobacco consumption behavior change during the COVID-19 pandemic is associated with perceived COVID threat**

**Authors & Affiliations:** Hollyann Loui, MD^1^; Joshua Li, BS^2^; Nicholas J. Jackson, PhD, MPH^2^; Ruby Romero, BA^3^; Lauren E. Wisk, PhD^3,4 §^ Russell G. Buhr, MD, PhD^1,4,5 §^

1. Division of Pulmonary & Critical Care, David Geffen School of Medicine at the University of California, Los Angeles, Los Angeles, CA, USA
2. Department of Medicine Statistics Core, David Geffen School of Medicine at the University of California, Los Angeles, Los Angeles, CA, USA
3. Division of General Internal Medicine & Health Services Research, David Geffen School of Medicine at the University of California, Los Angeles, Los Angeles, CA, USA
4. Department of Health Policy and Management; Jonathan and Karin Fielding School of Public Health at the University of California, Los Angeles, Los Angeles, CA, USA
5. Center for the Study of Healthcare Innovation, Implementation, and Policy, Health Services Research & Development, Greater Los Angeles Veterans Affairs Healthcare System, Los Angeles, CA, USA

§ denotes co-senior authors

**Corresponding Author:** Russell Buhr, MD, PhD; Division of Pulmonary & Critical Care Medicine, David Geffen School of Medicine at the University of California, Los Angeles. 1100 Glendon Avenue, Suite 850, Los Angeles, CA 90024. Tel: 310-267-2614, Fax: 310-794-0732, E-mail: [rbuhr@mednet.ucla.edu](about:blank)

**Supplemental Appendix Tables:**

**Supplemental Table 1. Change in smoked tobacco use based on perceived threat of COVID with inverse probability weighting for complete responses**

| N =113 | Model 1 | | Model 2 | | Model 3 | | Model 4 | | Model 5 | |
| --- | --- | --- | --- | --- | --- | --- | --- | --- | --- | --- |
| Smoking Change | **Relative Risk**  **(95% CI)** | **P-value** | **Relative Risk**  **(95% CI)** | **P-value** | **Relative Risk**  **(95% CI)** | **P-value** | **Relative Risk**  **(95% CI)** | **P-value** | **Relative Risk (95% CI)** | **P-value** |
| COVID Threat Scale Score (per SD change above mean) |  |  |  |  |  |  |  |  |  |  |
| *More v No Change* | 1.97  (1.03, 3.75) | 0.04 | 1.97  (1.03, 3.74) | 0.04 | 1.78  (0.91, 3.47) | 0.09 | 1.70  (0.89, 3.23) | 0.108 | 1.97  (1.03, 3.74) | 0.93 |
| *Less v No Change* | 1.78  (0.93, 3.40) | 0.08 | 1.78  (0.93, 3.41) | 0.08 | 1.62  (0.84, 3.1) | 0.15 | 1.69  (0.88, 3.24) | 0.114 | 1.78  (0.93, 3.41) | 0.08 |
| Age (per year change) |  |  |  |  |  |  |  |  |  |  |
| *More v No Change* |  |  | 1.00  (0.96, 1.04) | 0.90 | 1.02  (0.97, 1.06) | 0.46 | 1.01  (0.97, 1.06) | 0.55 | 1.00  (0.96, 1.04) | 0.90 |
| *Less v No Change* |  |  | 0.99  (0.95, 1.04) | 0.73 | 1.01  (0.96, 1.05) | 0.76 | 1.00  (0.96, 1.05) | 0.98 | 0.99  (0.95, 1.04) | 0.73 |
| PHQ-2 Score (per point change) |  |  |  |  |  |  |  |  |  |  |
| *More v No Change* |  |  |  |  | 1.67  (0.98, 2.82) | 0.057 |  |  |  |  |
| *Less v No Change* |  |  |  |  | 1.25  (0.72, 2.18) | 0.422 |  |  |  |  |
| GAD-2 Score (per point change) |  |  |  |  |  |  |  |  |  |  |
| *More v No Change* |  |  |  |  |  |  | 1.56  (0.93, 2.63) | 0.09 |  |  |
| *Less v No Change* |  |  |  |  |  |  | 1.32  (0.75, 2.34) | 0.34 |  |  |
| Anxiety or depression positive screen (either) |  |  |  |  |  |  |  |  |  |  |
| *More v No Change* |  |  |  |  |  |  |  |  | 2.75  (0.70, 10.79) | 0.15 |
| *Less v No Change* |  |  |  |  |  |  |  |  | 0.56  (0.16, 2.00) | 0.37 |

**Supplemental Table 2: Change in vaping behavior based on perceived threat of COVID with inverse probability weighting for complete responses**

| N =87 | Model 1 | | Model 2 | | Model 3 | | Model 4 | | Model 5 | |
| --- | --- | --- | --- | --- | --- | --- | --- | --- | --- | --- |
| Vaping Change | **Relative Risk**  **(95% CI)** | **P-value** | **Relative Risk**  **(95% CI)** | **P-value** | **Relative Risk**  **(95% CI)** | **P-value** | **Relative Risk**  **(95% CI)** | **P-value** | **Relative Risk (95% CI)** | **P-value** |
| COVID Threat Scale Score (per SD change above mean) |  |  |  |  |  |  |  |  |  |  |
| *More v No Change* | 0.96  (0.38,2.41) | 0.93 | 0.95  (0.38, 2.42) | 0.92 | 0.84  (0.32, 2.16) | 0.71 | 0.83  (0.30, 2.25) | 0.71 | 0.95  (0.38, 2.42) | 0.92 |
| *Less v No Change* | 1.67  (0.65, 4.29) | 0.29 | 1.64  (0.64, 4.20) | 0.30 | 1.61  (0.57, 4.50) | 0.37 | 1.87  (0.69, 5.07) | 0.22 | 1.64  (0.64, 4.20) | 0.30 |
| Age (per year change) |  |  |  |  |  |  |  |  |  |  |
| *More v No Change* |  |  | 1.00  (0.94, 1.05) | 0.88 | 1.00  (0.94, 1.07) | 0.91 | 1.00  (0.95, 1.06) | 0.94 | 1.00  (0.94, 1.05) | 0.88 |
| *Less v No Change* |  |  | 1.01  (0.96, 1.06) | 0.64 | 0.99  (0.94, 1.05) | 0.84 | 1.01  (0.96, 1.06) | 0.77 | 1.01  (0.96, 1.06) | 0.64 |
| PHQ-2 Score (per point change) |  |  |  |  |  |  |  |  |  |  |
| *More v No Change* |  |  |  |  | 0.98  (0.54, 1.78) | 0.95 |  |  |  |  |
| *Less v No Change* |  |  |  |  | 0.82  (0.46, 1.44) | 0.48 |  |  |  |  |
| GAD-2 Score (per point change) |  |  |  |  |  |  |  |  |  |  |
| *More v No Change* |  |  |  |  |  |  | 1.41  (0.57, 3.50) | 0.46 |  |  |
| *Less v No Change* |  |  |  |  |  |  | 0.69  (0.32, 1.50) | 0.35 |  |  |
| Anxiety or depression positive screen (either) |  |  |  |  |  |  |  |  |  |  |
| *More v No Change* |  |  |  |  |  |  |  |  | 1.65  (0.32, 8.38) | 0.55 |
| *Less v No Change* |  |  |  |  |  |  |  |  | 1.01  (0.25, 4.13) | 0.99 |

**Supplemental Table 3: Change in smoked tobacco use based on perceived threat of COVID u*sing multiple imputation***

| N =150 | Model 1 | | Model 2 | | Model 3 | | Model 4 | | Model 5 | |
| --- | --- | --- | --- | --- | --- | --- | --- | --- | --- | --- |
| Smoking Change | **Relative Risk**  **(95% CI)** | **P-value** | **Relative Risk**  **(95% CI)** | **P-value** | **Relative Risk**  **(95% CI)** | **P-value** | **Relative Risk**  **(95% CI)** | **P-value** | **Relative Risk (95% CI)** | **P-value** |
| COVID Threat Scale Score (per SD change above mean) |  |  |  |  |  |  |  |  |  |  |
| *More v No Change* | 1.68  (1.06, 2.66) | 0.026 | 1.72  (1.07, 2.77) | 0.025 | 1.71  (1.00, 2.93) | 0.049 | 1.59  (0.97, 2.61) | 0.064 | 1.76  (1.07, 2.87) | 0.025 |
| *Less v No Change* | 1.77  (1.07, 2.94) | 0.026 | 1.73  (1.06, 2.83) | 0.028 | 1.74  (1.06, 2.85) | 0.029 | 1.72  (1.05, 2.83) | 0.032 | 1.73  (1.05, 2.86) | 0.030 |
| Age (per year change) |  |  |  |  |  |  |  |  |  |  |
| *More v No Change* |  |  | 0.98  (0.95, 1.00) | 0.094 | 1.00  (0.97, 1.02) | 0.756 | 1.00  (0.97, 1.03) | 0.814 | 0.99  (0.96, 1.02) | 0.464 |
| *Less v No Change* |  |  | 1.01  (0.99, 1.04) | 0.294 | 1.01  (0.99, 1.05) | 0.327 | 1.02  (0.99, 1.05) | 0.219 | 1.01  (0.98, 1.04) | 0.421 |
| PHQ-2 Score (per point change) |  |  |  |  |  |  |  |  |  |  |
| *More v No Change* |  |  |  |  | 1.63  (1.24, 2.14) | <.001 |  |  |  |  |
| *Less v No Change* |  |  |  |  | 1.00  (0.77, 1.30) | 0.970 |  |  |  |  |
| GAD-2 Score (per point change) |  |  |  |  |  |  |  |  |  |  |
| *More v No Change* |  |  |  |  |  |  | 1.52  (1.21, 1.92) | <.001 |  |  |
| *Less v No Change* |  |  |  |  |  |  | 1.09  (0.85, 1.39) | 0.488 |  |  |
| Anxiety or depression positive screen (either) |  |  |  |  |  |  |  |  |  |  |
| *More v No Change* |  |  |  |  |  |  |  |  | 3.59  (1.40, 9.21) | 0.007 |
| *Less v No Change* |  |  |  |  |  |  |  |  | 0.72  (0.29, 1.81) | 0.490 |

**Supplemental Table 4: Change in vaping behavior based on perceived threat of COVID u*sing multiple imputation***

| N =110 | Model 1 | | Model 2 | | Model 3 | | Model 4 | | Model 5 | |
| --- | --- | --- | --- | --- | --- | --- | --- | --- | --- | --- |
| Vaping Change | **Relative Risk**  **(95% CI)** | **P-value** | **Relative Risk**  **(95% CI)** | **P-value** | **Relative Risk**  **(95% CI)** | **P-value** | **Relative Risk**  **(95% CI)** | **P-value** | **Relative Risk (95% CI)** | **P-value** |
| COVID Threat Scale Score (per SD change above mean) |  |  |  |  |  |  |  |  |  |  |
| *More v No Change* | 1.23  (0.68, 2.23) | 0.490 | 1.22  (0.67, 2.23) | 0.518 | 1.19  (0.63, 2.24) | 0.584 | 1.01  (0.51, 2.00) | 0.982 | 1.15  (0.61, 2.18) | 0.668 |
| *Less v No Change* | 2.08  (1.05, 4.12) | 0.035 | 2.05  (1.04, 4.04) | 0.037 | 2.04  (1.05, 3.97) | 0.034 | 2.14  (1.08, 4.22) | 0.028 | 2.09  (1.07, 4.08) | 0.030 |
| Age (per year change) |  |  |  |  |  |  |  |  |  |  |
| *More v No Change* |  |  | 0.99  (0.95, 1.02) | 0.474 | 0.99  (0.96, 1.03) | 0.632 | 1.00  (0.96, 1.03) | 0.831 | 0.99  (0.96, 1.03) | 0.609 |
| *Less v No Change* |  |  | 1.01  (0.98, 1.04) | 0.403 | 1.01  (0.98, 1.04) | 0.526 | 1.01  (0.98, 1.04) | 0.528 | 1.01  (0.98, 1.04) | 0.512 |
| PHQ-2 Score (per point change) |  |  |  |  |  |  |  |  |  |  |
| *More v No Change* |  |  |  |  | 1.19  (0.90, 1.58) | 0.217 |  |  |  |  |
| *Less v No Change* |  |  |  |  | 0.90  (0.68, 1.17) | 0.417 |  |  |  |  |
| GAD-2 Score (per point change) |  |  |  |  |  |  |  |  |  |  |
| *More v No Change* |  |  |  |  |  |  | 1.33  (0.99, 1.80) | 0.058 |  |  |
| *Less v No Change* |  |  |  |  |  |  | 0.90  (0.69, 1.16) | 0.416 |  |  |
| Anxiety or depression positive screen (either) |  |  |  |  |  |  |  |  |  |  |
| *More v No Change* |  |  |  |  |  |  |  |  | 1.93  (0.66, 5.65) | 0.231 |
| *Less v No Change* |  |  |  |  |  |  |  |  | 0.64  (0.24, 1.66) | 0.354 |
